# B cell immunity and therapeutic opportunities in brain metastases

**DOI:** 10.3389/fimmu.2025.1740386

**Published:** 2026-01-14

**Authors:** Grace Jones, Alina Murphy, Catalina Lee-Chang

**Affiliations:** 1 Department of Neurological Surgery, Feinberg School of Medicine, Northwestern University, Chicago, IL, United States; 2 Malnati Brain Tumor Institute of the Lurie Comprehensive Cancer Center, Feinberg School of Medicine, Northwestern University, Chicago, IL, United States

**Keywords:** B cells, brain metastasis, brain tumor, immune microenvironment, immunotherapy

## Abstract

Arising from extracranial cancers, brain metastases (BrM) are the most prevalent brain malignancy in adults. Even though there are recent advances in systemic cancer therapies and immunotherapies, the prognosis for BrM remains poor, with median survival rather dismal. The central nervous system (CNS) presents a distinct immunological and structural landscape that restricts immune surveillance and effective therapeutic delivery. This immune privilege is enforced by the blood brain barrier (BBB), specialized myeloid populations, in conjunction with reduced lymphatic drainage, which collectively constrains the effector immune cell trafficking and antigen presentation. Thus, immunotherapeutic strategies that have revolutionized systemic oncology, such as immune checkpoint inhibitors (ICIs) and chimeric antigen receptor (CAR)-T cell therapies, have presented only rather modest benefit in BrM. While immunotherapy-focused research has largely focused on T-cell-mediated mechanisms, an accumulation of recent findings suggest that B cells play multifaceted and underexplored roles within the unique CNS tumor microenvironment (TME). Aside from antibody production, B cells contribute to antigen presentation, cytokine secretion, and the formation of tertiary lymphoid structures which are functions that can either promote or suppress antitumor immunity depending on their differentiation state and local cues. In primary brain tumors, like glioblastoma (GBM), B cell infiltration has been linked to both enhance immune activation and immune regulation, yet their significance in BrM remains comparatively undefined. Understanding how B cells adapt and function within the niche constraints of the CNS, such as how they influence immune suppression, antigen presentation, and TME remodeling, may reveal new therapeutic vulnerabilities and allow for harnessing complementary B cell-based immunotherapies instead of T cell-focused approaches. This review synthesizes current knowledge on the structural and immunological features that differentiate BrM from primary brain tumors and extracranial metastases. We highlight the emerging evidence on B cell biology in the CNS, and discuss their immunostimulatory and immunoregulatory capacities, while exploring ongoing efforts to leverage B cell-based immunotherapies in brain malignancies, specifically proposed BrM. By defining the immunological landscape of BrM and the therapeutic promise of B cells, this work suggests a new possibility in CNS oncology, where humoral immunity may be harnessed to target brain metastatic malignancies.

## 1 Introduction

Brain metastases (BrM) represent the most common intracranial tumors in adult cancer patients, stemming from extracranial malignancies. There is a higher incidence of metastasis to the brain in lung, breast, and melanoma primary cancers compared to other cancer forms (1, 2). Despite advances in systemic cancer therapies, the prognosis for patients with BrM remains unfortunate, with a median survival of only several months (3). Due to the unique nuances of the brain’s composition, the presence of the blood-brain barrier (BBB), and reduced immune infiltration, drug penetration and effective immune surveillance are limited, presenting restrictions for therapy options (4, 5). Treatment approaches commonly include surgery, radiotherapy (often whole-brain), or stereotactic radiosurgery, although survival remains dismal. Recent evidence suggests systemic therapy, either targeted agents or immunotherapy, can be effective in combating BrMs, especially those originating from well-characterized primary tumors (6).

The establishment of metastatic cancer encompasses entering the central nervous system (CNS) from the blood and crossing the BBB (7). From there, clonal tumor cells proliferate and invade locally, causing tissue displacement, inflammation, and edema (1). The tightly regulated immune environment of the brain differs from that of peripheral tissues. The distinct immunological landscape of the brain is characterized by selective infiltration of T and B lymphocytes, specialized myeloid populations, and limited lymphatic trafficking, all of which exemplify the brain’s distinct immune context (8). These features present particular complexities with immunotherapies and suggest the need for specific approaches tailored to brain metastases (9). However, immunotherapy has recently revolutionized treatment management, particularly for systemic metastases, specifically melanoma-derived BrM (10). Treatment with immune checkpoint inhibitors (ICIs) in BrM patients has shown some success with improvement of median survival, but efficacy remains inconsistent, primarily due to the immunosuppressive microenvironment (11, 12).

Recent accumulating evidence suggests that B cells play a multifaceted and relatively underexplored role in shaping the BrM tumor microenvironment (TME). Aside from their canonical functions in antibody production and cytokine secretion, B cells also contribute to antigen presentation and regulation of other immune populations (13, 14). B cells can organize into tertiary lymphoid structures (TLS) within or adjacent to tumor sites, which have been linked to enhanced tumor surveillance, improved response to immunotherapy, and favorable prognosis in several solid tumors, such as melanoma, lung, and breast cancers (15–17). While TLS are well-characterized and defined in peripheral tumors, their presence, architecture, and functional relevance in BrM remain poorly defined, which represents an important knowledge gap and potential avenue for therapeutic intervention. While ICIs and chimeric antigen receptor (CAR) T- cell therapies show promise in peripheral tumors, the y aren’t particularly successful in BrM treatment due to the suppression of immune-mediated targeting within the TME and poor trafficking within the CNS (18). This synthesis defines the novelty of this review compared to what is previously established regarding TLS and B cells in solid tumors by focusing on the confinements of the CNS and considerations of leveraging a different lens for BrM treatment.

While significant focus is placed on T cell-based immunotherapy strategies, these often fail due to poor trafficking and immune escape, suggesting the need for exploration of alternative immune modulators. B cell-based therapies, in the preclinical setting, have shown success in primary brain tumors (19) and could be an avenue for promising therapy in malignant brain metastases that could overcome the limitations of T cell-centric approaches. The unique biology and characteristics of B cells allow for antigen presentation, antibody production, and cytokine secretion in combination with regulating immune response, exemplifying the multifaceted role of B cells in antitumor activity (20, 21). In this review, we will discuss the current immunotherapeutic landscape for BrM and discuss the possibility of harnessing the unique functions of B cells to propose future utilization for cancer immunotherapy.

## 2 Why brain metastases are different

Differing markedly from primary brain or extracranial tumors, BrM by nature are more nuanced, compartmentalized, and influenced by the CNS. The brain is sequestered anatomically and is immunologically shaped by the unique microenvironment, influencing tumor proliferation tendencies, response to treatment, and interaction with host immunity. To better understand how BrM navigate the CNS and how B cells modulate the metastatic progression, the following sections will preface and further examine the structural, cellular, and unique immunological features of the brain that contribute to metastatic colonization and therapeutic responses or barriers.

### 2.1 Brain metastasis formation

Brain metastasis formation includes a highly selective, multistep process where circulating tumor cells (CTCs) must withstand hematogenous dissemination as they leave the primary tumor and enter blood circulation, arrest in the cerebral microvasculature, and traverse the BBB (22, 23). Following intravasation from the primary tumor, CTCs become sequestered in brain capillaries, and here the endothelial interactions and mechanical constraints determine their initial lodgment (22). For successful extravasation, tumor cells must engage and disrupt the specialized BBB architecture, which is filled with endothelial cells connected via tight junctions, in addition to pericytes, astrocytic end-feet, and basement membrane, that all collectively allows for CNS homeostasis, as discussed later in this section (24). Tumor-derived proteases, inflammatory cytokines, and exosomes can destabilize endothelial junctions, which reshapes the perivascular niche and aids in creating a heterogeneous blood-tumor barrier (BTB) that differs in permeability across metastases but is rather leaky in nature (25). The variability and disruption of the BBB is now better understood, with some BrMs maintaining an intact barrier while others exhibit substantial leakage. This notable heterogeneity impacts early BrM colonization, immune infiltration, and therapeutic penetration and potential (25, 26).

Upon entering the brain parenchyma, metastatic cells often co-opt existing vasculature, such as vessels and endothelial-astrocytic signals, to support initial survival and aid in motility (27, 28). Reactive astrocytes initially produce cytotoxic responses but transition quickly to a tumor-suppressive phenotype through cytokine secretion, like interleukin-6 (IL*-*6), interleukin-10 (IL-10) and transforming growth factor-beta (TGF-β). This promotes immune evasion and micrometastatic outgrowth (29–31). Microglia and infiltrating macrophages shape a permissive niche by releasing immunosuppressive mediators and remodeling extracellular matrix (ECM) structures (29).

Emerging evidence suggests that B cell dynamics further modulate these latter metastatic stages. In BrM particularly, B cells display functional heterogeneity. This is seen in variable functions including antigen-presenting and antibody-producing subsets with antitumor capacity, in addition to regulatory B cells (Bregs) which amplify IL-10-driven immunosuppression and inhibit T cell activity (21, 22). B cells also notably organize into tertiary lymphoid structures, which variably promote immune activation, or support metastatic progression depending on their respective cellular composition and cytokine profile (32, 33). Antibody-secreting B cells may also influence BrM growth by modulating complement pathways such as Fc-receptor signaling and myeloid polarization. Overall, the multitude of B cell functions, duality of responses, and mediation of antibody activation and presentation, cytokine signaling, and role in cross talk of processes together with blood vessels of the brain and support cells either facilitate the progression of small clustering to overt brain metastases, or conversely, promote anti-tumor immunity (23, 30, 33).

### 2.2 B cell differentiation pathways as determinants of anti- and pro-tumor function in BrM

Circulating B cells, which tend to be largely naïve and classical memory subsets, differ functionally and phenotypically from tumor-infiltrating B cells, which are recognized to undergo local activation, clonal expansion, and differentiation within the TME (34, 35). Naïve (have not encountered a specific antigen) and memory cells (long-lived, antigen-specific) have distinct activation thresholds and antigen-presenting capacities that allow memory cells a quicker transition to antibody responses upon antigen re-exposure (36, 37). In solid tumors, and including BrM, infiltrating B cells tend to adopt class-switched memory or plasmablast/plasma cell phenotypes and can organize into TLS-like structures that perform local antigen presentation and promote vital T cell priming (34, 38, 39). Functionally opposed to this are Bregs, as mentioned above, which produce IL-10, TGF-β, or express PD-L1 and hinder effector T cells and dendritic cell functions (21, 22). Tumor-derived factors and stromal cues (i.e., IL-6, prostaglandins, CNS-specific astrocyte or microglial signaling) can promote Breg differentiation or expansion, thereby favoring local immunosuppression (40, 41). A combination of clinical and mechanistic evidence has suggested that a balance among these B cell subpopulations determines if B cells support anti-tumor immunity through antigen presentation and antibody production, or whether they impede anti-tumor immunity via Breg-mediated suppression. Thus, the phenotypic characterization of B cells is essential for potential prognostic and applications of B cell therapeutics (42).

### 2.3 Immune and stromal programs shaped by primary tumor of origin

In comparing BrM formation arising from different primary tumor types, it is important to recognize the tumor-origin-dependent immune and stromal programs (astrocyte-mediated processes in this case) that share similar properties while also diverging from the primary malignancy (43–45). Multiple cohorts and mechanistic studies have shown that lung- and melanoma-derived BrM tend to retain higher lymphoid activity, such as B and T cells and TLS signatures, in addition to immune-checkpoint pathway expression, whereas breast cancer-derived BrM are often more myeloid and neutrophil dominated with noted lymphoid scarcity (43, 45, 46). These patterns aid in explaining the varying responsiveness to immunotherapies across primary cancers (see clinical series and meta-analyses; JCO ASCO abstract) (44, 45). Recent immunoprofiling and spatial transcriptomic analyses have revealed distinct immune-stromal niches and TLS-associated programs enriched in lung and melanoma BrM, but downregulated in breast cancer-derived BrM (46, 47). From a mechanistic perspective, tumor cells from lung and breast primaries exploit CNS-specific stromal interactions, such as astrocyte-mediated STAT3 and survival signaling (e.g., BCL2L1. TWIST1, GSTA5 as discussed further in section 2.2.1), in order to promote colonization and therapy resistance within the brain microenvironment (43, 48). Overall, this highlights the primary-origin influence and brain-adaptation signaling that shape BrM biology and formation. Thus, therapeutic strategies should be CNS-specific and tailored to the tissue of origin to overcome shared stromal limitations.

### 2.4 Blood brain barrier and immune privilege

The concept of CNS immune privilege emerged from experimental pursuits in the mid-20^th^ century (22). These were transplantation studies that showed grafts survived longer in the brain compared to peripheral sites, suggesting that the brain was immunologically unique (49). This apparent tolerogenic nature is conditional, because it remains vulnerable to activated peripheral immunity, where effector cells can further infiltrate the CNS. The immune privilege of the CNS arises from and is associated with structural and functional barriers. One distinguishing feature includes the BBB, as mentioned previously (50). Specifically, the BBB is formed by endothelial cells lining the cerebral microvasculature, interconnected by tight junctions, and supported by pericytes and astrocytic end-feet (51). Within the neurovascular unit, astrocytes exhibit a dual role which entails the initial release of neuroinflammatory mediators to restrict metastatic growth, but later shift toward a tumor-supportive phenotype by secreting growth factors and immunosuppressive cytokines, including TGF-β and IL-10 (52, 53). Through these cellular interactions, the BBB functions as an interface that regulates trafficking between the bloodstream and brain parenchyma on a molecular and cellular level. For this reason, the “barrier” presents a dual challenge in the BrM context. First, the BBB rigidly controls molecular agents and cellular immunotherapies, especially the delivery of antibodies or other immune modulators. In addition, the BBB limits immune cell infiltration, which leads to the development of an immune-privileged site where tumor cells can evade immune surveillance. The permeability of the BBB appears selectively variable (tumor, region, and molecule-specific) and heterogeneous. Some regions are impermeable, while others allow for selective penetration of therapeutics. Unlike glioblastoma (GBM), where neovascularization produces malformed and leaky vessels, the extent of neovascularization in BrM is less defined and may vary by tumor origin type and model system. This variability in BBB integrity fosters a spatial heterogeneity in immune cell infiltration and functioning throughout BrMs and presents complications for the efficacy and delivery of treatments. Notably, it appears that effects of brain metastasis on the leakiness of the BBB is cell line-dependent in preclinical models, where BBB can remain intact or are in a disrupted (50, 54, 55).

In the BrM context, the overall neurovascular unit interactions are significantly altered. Disruptions within the neurovascular interface are of concern because perturbations may promote tumor cell entry and reshaping of the respective microenvironment that could contribute to metastatic growth (56).

### 2.5 CNS immune landscape and infiltrating immune cells

The brain’s immune landscape is distinguished by its dependence on resident immune cells and selective restriction of peripheral immune entry. Microglia and astrocytes constitute the main immune sentinels, although their activated states substantially differ compared to those of peripheral macrophages (57). In the periphery, macrophages tend to polarize along a spectrum between a pro-inflammatory state (M1), or a more anti-inflammatory, tissue-repairing state (M2). In CNS malignancies and BrM, microglia commonly induce a pro-tumor and anti-inflammatory, M2-like response, which hinders cytotoxic T cell recruitment and effector function (58). Responsible for this hindrance are the activated microglia and astrocytes which secrete immunosuppressive mediators such as IL-10 and TGF-β1. Unlike peripheral macrophages, CNS-resident myeloid cells are tightly associated with the neurovascular unit, where they aid in local inflammation regulation while also enforcing immune privilege by dampening effector responses.

Among the secreted mediators, TGF-β1 has emerged as a central orchestrator of immunosuppression in brain tumors. TGF-β1 is a modulator of proliferation, ECM deposition, and immune balance through canonical and non-canonical pathways (59, 60). It plays a context-dependent role in BrM, as it can facilitate a pro- or anti-tumor response. A notable role of TGF-β that amplifies the migratory and invasive tendencies of cancer is also suggested with it being an inducer of the epithelial-mesenchymal transition (EMT), where it suppresses immune cells in the brain, allowing for tumor cells to cross the BBB and enable metastatic progression. Its effects are context dependent because as it inhibits effector T cell proliferation and sustains the function of Tregs, it can simultaneously recruit macrophages, and in combination with IL-6, promote Th17 differentiation (59–61). As a result, this produces considerable amounts of IL-17 and maintains acute inflammation. In gliomas (primary glial-derived brain tumors), malignant cells exploit TGF-β1 in order to drive mesenchymal transition, invasion, and stem-like self-renewal. In BrM, astrocyte-derived TGF-β1 suppresses cytotoxic CD8**^+^** T cell function and supports metastatic growth (62). Importantly, TGF-β1 also shapes B cell behavior within the BrM microenvironment (63, 64). As preliminarily suggested in recent work, spatial transcriptomic analyses in GBM show that myeloid cells are major sources of TGF-β1 activity within intratumoral B cell niches, suggesting that myeloid-B cell interactions are an important modulator of B cell suppression (64). This myeloid-driven TGF-β1-mediated suppression likely influences the regulatory B cell phenotype observed, which limits their capacity to stimulate T cells and form functional TLS (63, 64). Thus, as immunotherapies largely focus on targeting T cells, B cells in the TME are also impacted by TGF-β-dependent immunosuppression, which could halt APC function and hinder immune response (63, 65). Hou et al. recently demonstrated that dual blockade of αVβ8 integrin (activates TGF-β) and PD-1 restores B cell function, expands intratumoral B cells, and synergizes with PD-1 inhibition in order to eradicate nearly 60% of treated mice (64). This dual therapy was also shown to reduce B cell-mediated suppression of CD8**^+^** T cell cytotoxicity and promote antitumor B cell activity (64). These findings underscore TGF-β as a key regulator of B cell proper functioning within CNS tumors and suggests that overcoming TGF-β-mediated B cell suppression could be critical in BrM, where there are similar myeloid-predominant niches and patterns of immune exclusion (64, 66). Clinical studies, involving patients with high-grade gliomas and brain metastases, further underscore the relevance of this mediator. For example, functional *TGFB1* promoter polymorphisms correlate with greater circulating TGF-β1, adverse clinical features, and dismal survival trajectory in patients with brain malignancies (59). Overall, these findings position TGF-β1 as both a mechanistic facilitator of immune evasion and a possible prognostic predictor within the CNS tumor microenvironment, with its impact dependent on the context and possible tumor type.

### 2.6 BrM vs. extracranial metastases and primary brain tumors

BrM stem from peripheral cancers and overcome the structural and immunological barriers of the CNS in order to establish in the brain. The CNS imposes barriers to both immune trafficking and antigen flow, BBB and blood-cerebrospinal fluid (CSF), and has a distinct stromal milieu (astrocytes, microglia, perivascular macrophages) which shapes spatial patterns of immune exclusion that profoundly alter immune-tumor interactions (67, 68). By contrast, extracranial metastases arise in tissues with abundant lymphatic drainage and ongoing immune system influence, which allows for antigen presentation and T-cell priming. This means that BrM retain properties of their tissue of origin, they also rewire to survive within a more constrained immune landscape (69). This unique immune cell composition and spatial organization are two reasons why therapeutic responses in BrM diverge markedly from responses of corresponding extracranial tumors.

Brain metastases represent the product of convergent pressures imposed by the CNS microenvironment. In particular, melanoma brain metastases (MBM) highlight this principle, especially as representative in the work of Izar et al (70). MBM display pronounced chromosomal instability, micronuclei formation, and with notable neuronal-like differentiation programs (NGFR, NCAM1, NLGN3), which facilitate integration into neural circuits (70). MBM cells preferentially engage oxidative phosphorylation and PI3K/ERBB signaling, while also maintaining melanocytic lineage activity. This combination distinguishes this derivation of BrM from extracranial melanoma metastases (ECM), which instead favor EMT, AXL-drive invasive states (causes amplified invasiveness and drug resistance), and mTORC1 enrichment (70). Several of these features are also seen in other derivations of BrM, like breast and lung brain metastases. In these forms, there is also notable chromosomal instability, neuronal mimicry, and reliance on oxidative phosphorylation, which showcases the convergent metabolic and transcriptional alterations that support colonization of the neural niche. Across diverse BrM, shifting of the immune landscape toward monocyte-derived macrophages and the high expression of TOX (TOX**^+^**CD8**^+^** T cells, associated with T cell dysfunction) appears to be a shared tendency (38). Compared to breast and lung BrM, melanoma BrM have robust lymphoid aggregates with evidence of B cell-to-plasma cell maturation (70, 71).

**Figure 1:**
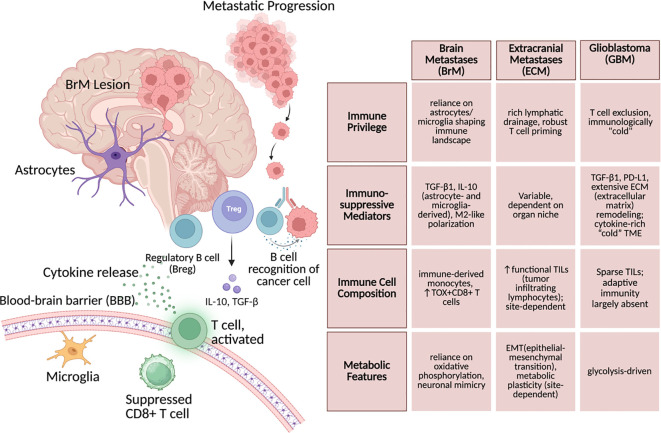
Brain metastasis lesion detailed with surrounding immune environment, with comparisons between BrM and extracranial metastases and glioblastoma.

In contrast, GBM. the most common malignant primary brain tumor, diverges markedly from these metastatic developments and environments (72). Rather than adopting neuronal mimicry, GBM arises from neural precursor-derived stem-like populations and is sustained by transcriptional programs of plasticity (like SOX2, OLIG2, NANOG) that emphasize its proneural, mesenchymal, and classical subtypes (73, 74). Core drivers include PDGFRA/IDH1 alterations, NF1 loss, and EGFR amplification, which fuels Akt/STAT3 and NF-κB signaling pathways. Unlike BrM, GBM favors glycolysis, and thrives within the compact brain parenchyma, where extracellular space is limited. Glioblastoma stem cells (GSCs) occupy perivascular niches, where stromal and mesenchymal elements reinforce the “stem-like” state through activation of Wnt/Notch signaling and proteolytic remodeling of the extracellular matrix (which facilitates invasion) (75). Over time, selective stress exerted by treatments tend to promote a shift from proneural to mesenchymal states, a transition that fosters recurrence and resistance. Immunologically, GBM epitomizes the “cold” CNS tumor. This entails the TME being dominated by microglia and infiltrating macrophages, while adaptive immunity is constrained by T cell exclusion and immunosuppression driven by tumor-stromal cross-talk (76). This entrenched stromal and immune landscape juxtaposes that of BrM, which, while constrained by the CNS, retains greater inflammatory potential and lineage-specific immune engagement (**Figure 1**).

## 3 Brain tumor microenvironment and BrM immunological tendencies

The brain TME reflects the intersection of CNS-resident cells, infiltrating immune cell populations, and mediators that influence tumor progression and therapy response, within an immune-privileged setting that regulates lymphocyte trafficking and function, as previously discussed (77). Although not immunologically inert, the BrM niche has dynamic and variable immune activity that fuels the heterogeneity of cancer cells, clonal evolution, and therapeutic resistance, which diverges from that of primary brain malignancies. These distinctions are vital to understand the reasoning behind proposed treatment strategies and targets for BrM (78).

### 3.1 BrM microenvironment cancer-specific findings

BrMs vary cellularly and molecularly from primary brain tumor developments. In BrM derived from different primary tumors such as lung, breast, and melanoma, there are notable differences in immune landscapes. BrM derived from lung cancer have higher expression of commonly mutated genes, particularly in genes that regulate immune checkpoint pathways, such as tumor necrosis factor receptor superfamily 9 (TNFRSF9), tumor necrosis factor receptor superfamily 4 (TNFRSF4), programmed cell death 1 ligand 2 (PDCDILG2), indoleamine 2,3-dioxygenase 1 (IDO1), inducible T cell costimulator (ICOS), and more (78). This suggests that lung cancer BrM could be increasingly sensitive or responsive to immunotherapy, compared to other BrM forms (78, 79). On the other hand, BrM stemming from melanoma tend to have a higher presence of CD4**^+^** and CD8**^+^** T cells, while breast cancer-derived BrM has a greater incidence of neutrophils and macrophages. This could suggest potential increased therapeutic resistance of breast cancer BrM juxtaposed to melanoma BrM (69, 78, 80). Lung cancer BrM has also been noted to have more T lymphocytes while melanoma has more B lymphocytes. Despite several differences between different microenvironments of BrMs derived from varying cancer forms, there remain similarities as well. As supported by *in vivo* experiments, both breast and lung cancer cells interact with astrocytes in the CNS in order to bolster the expression of survival genes, such as B cell lymphoma-2-like protein 1 (BCL2L1), twist family BHLH transcription factor 1 (TWIST1), and glutathione S-transferase alpha 5 (GSTA5) to acquire drug or therapy resistance (78, 81, 82). The variations in BrM microenvironments compared to those of primary brain malignancies provide important insights for treatment options, such as cancer-origin-dependent differences or mechanistic adaptation in the CNS, which necessitate therapeutic strategies tailored to BrM.

### 3.2 Novel mechanistic insights into intracranial TLS and B cell immunity

Foundational work in melanoma studies and other solid tumors has provided evidence that TLS and mature B cell states robustly correlate with response to immune checkpoint blockade (ICB). For instance, Helmink et al. demonstrated that activated and memory B cells within TLS predict ICB responses in melanoma (83), while Cabrita et al. showed that TLS density stratifies survival and therapeutic benefit (84). Similarly, Narvaez et al. suggested that TLS presence in breast cancer predicts improved survival and higher pathological complete response rates (85). These findings in conjunction with evidence suggesting B cells drive local antigen presentation, class switching, and CD4/CD8 T cell coordination contribute to a substantial framework for understanding how B cell immunity supports anti-tumor responses in extracranial sites (86).

Implications for BrM arise when these findings are juxtaposed with the niche constraints of the CNS. Although recent spatial and single-cell evidence has revealed that the presence of B cell aggregates and TLS-like structures in breast and lung-derived BrM, these intracranial TLS often exhibit incomplete organization, hindered germinal-center maturation, and altered chemokine expression (87). BrM studies have shown diminished levels of the B cell chemoattractant CXCL13, increased microglia and astrocyte-related suppression, and lymphocyte entry restrictions due to the BBB (84, 88). These features could limit the development of entirely functional TLS, thereby reducing the potential of B cells to harness ICB responses compared to melanoma or breast cancer in peripheral sites. Relevant findings from Mughal et al. have shown TLS signatures, which are observed in one-third of BrM and most frequent in lung cancer and melanoma metastases, mark tumors with strong B cell and T cell activation, including high immunoglobulin gene expression, mediated T cell recruitment, and lymphotoxin beta (LTß) upregulation (89). TLS-positive BrM also contains organized lymphoid aggregates, and specifically noted in lung cancer BrM patients, are strongly associated with prolonged survival, which underscores their relevance as a marker in BrM (89). Overall, the variance of cellular components of the tumor immune microenvironment in BrM is dependent on cancer type, and signify a key role of TLS formation for prognostic relevance and outcome in BrM patients (89).

Recent analyses from Nohira et al. suggest that B cell-rich immune niches could tangentially serve as prognostic markers in BrM (90). In a cohort of lung cancer BrM samples, TLS-like aggregates and associated B cell phenotypes, such as class switched memory states and germinal center-like activity, were detected in a subset of patients and were strongly associated with prolonged survival. TLS density, rather than the overall density of tumor-infiltrating lymphocytes (TILs), was the immune variable positively and independently correlated with patient outcome (90). This parallels the understanding in extracranial tumors, where TLS predict improved response to immunotherapy and promising prognosis, but applies these principles to the intracranial context where functional relevance of B cell immunity has remained relatively undefined. These patterns and findings suggest that B cell activation within the brain is shaped by neuroinflammatory cues along with tumor antigens, delineating the intracranial TLS biology from well-characterized TLS in extracranial malignancies (83–85, 90).

Altogether, these fundamental and emerging studies underscore that BrM exhibit a markedly distinct, CNS-influenced B cell landscape that contributes to or reshapes antigen presentation, antibody maturation, and response to ICB. Understanding these key differences between BrM and extracranial primary tumors suggests why BrM often respond poorly to current immunotherapies and presents a need for brain-specific strategies in order to induce functional TLS, enhance B cell recruitment and activation, and ultimately overcome neuroimmune suppression (89).

## 4 Immune escape mechanisms

Through a convergence of tumor-intrinsic signaling and microenvironmental reprogramming, brain metastases evade immune surveillance. A central axis involves the upregulation of immune checkpoints (PD-L1, LAG-3, TIGIT), which induces T cell exhaustion despite the relatively high TIL burden observed in melanoma and lung BrM (9). This somewhat differs from GBM, which is immunologically “cold,” as characterized by decreased T cell infiltration (91). Both gliomas and BrM exhibit dynamic interaction and crosstalk between tumor and immune cell populations, with varying drivers and contributors that aid in remodeling the TME (34, 92).

Cytokine-mediated suppression further dampens anti-tumor immunity. Both tumor cells and reactive astrocytes secrete factors such as TGF-β, IL-10, and MIF (macrophage migration inhibitory factor), which collectively inhibit cytotoxic T cell function and propel the formation of suppressive “astrospheres” (9). These astrospheres are suppressive of CD8**^+^** T cells in the brain, which results in immune evasion and the growth of BrM (9, 30). This immunoregulation works in conjunction with tumor-intrinsic adaptations. Disseminated tumor cells avoid being eradicated by the brain’s natural defenses, such as parenchymal cell surveillance and cytotoxic defenses, by producing serpins, which disable the Fas ligand (FasL)-induced apoptosis pathway, further allowing the tumor cells to persist and grow in the hostile brain environment. Interestingly, the BrM niche is primed by molecular cues for metastasis as well, which originate from extracellular vesicles (EVs). These EVs can be released from primary tumors and diverse tumor-associated stromal cells (microglia, astrocytes, fibroblasts, and endothelial cells), and fuel the migration of cancer cells across the BBB, promoting brain tropism while contributing to immune evasion (9, 93).

Kato et al. (Ref #95) suggest that EVs, specifically tumor-derived EVs (TDEs), are key regulators of antitumor immunity with significant influence on B cell activation, differentiation, and function (94). TDEs transport tumor-associated antigens (TAAs), MHC-peptide complexes, and costimulatory molecules that can directly engage B cells and seem to drive antigen uptake, activation, class switching, and antibody generation in B cells (94–96). EV-B cell interactions also shape the broader immune environment and network as EV-primed B cells enhance CD4**^+^** and CD8**^+^** T cell responses through antigen presentation and the release of key cytokines, which contributes to coordinated humoral and cellular immunity, (for further review, PMID 32580358) (94). Conversely, this review highlights that TDEs also can majorly suppress B cell immunity, acting as antibody decoys, transferring immunosuppressive ligands, such as HLA-G, and possibly pushing B cells toward Bregs which inhibit effector T cells (94). This further highlights the role of TDEs in promoting immunosuppression. Overall, the EV-B cell interplay functions as a bidirectional system that can either amplify or suppress anti-tumor immunity (94, 95). Evidence suggests distinct roles of EVs in the TME and systemically, a distinction that carries relevance for metastatic progression and for EVs capable of traversing to the brain (94). Considering the presence of B cells and Breg-like populations in BrM, EV-mediated phenotypes are relevant in metastasis to the CNS, where they may contribute to local immune suppression, TLS formation, and impact therapeutic potential in treating BrM.

In a recent spatial transcriptomics study of 44 non-small cell lung cancer BrM patients, Zhang et al. found considerable remodeling of the brain TME, such as enriched M2-type macrophages, reactive astrocytes, cancer-associated fibroblasts (CAFs), and ECM components, in conjunction with reduced infiltration and activation of T and B cells, decreased antigen-presentation signatures, and upregulation of immune-checkpoint and immunoregulatory genes despite fibrotic status (fibrotic vs. non-fibrotic brain TME) (97). These distinct fibrous subtypes exhibit notably differing immune landscapes. Fibrotic brain TME is dominated by the presence of immunosuppressive M2 favoring macrophages and ECM deposition, whereas the non-fibrotic brain TME displays increased expression of checkpoint molecules and immature microglia or astrocytes (97). This distinct spatial reprogramming within the TME supports the emergence of an immunosuppressive, fibrogenic metastatic niche, undermining adaptive immune responses (97, 98). Comparatively, spatial-omics analyses in gliomas, as characterized well by Yu et al., particularly GBM, reveal a different but still instructive spatial organization (99). Single-cell RNA-seq and spatial transcriptomics depict stratified metabolic and permissive margins, such as glycolytic, hypoxic tumor cores juxtaposed with more metabolically permissive margins, corresponding to spatially restricted immune cell infiltration, metabolic gradients, and suppression of lymphocyte activation (99). In gliomas, immune-related subpopulations such as tumor-associated macrophages (TAMs), regulatory T cells (Tregs), and other immune cells are differentially distributed where immune-rich regions tend to be localized to the periphery, reactive zones, or invasive margins (99). These findings suggest how metabolic reprogramming and spatial niche architecture in glioma influence immune composition and function (98–100).

In addition, myeloid cell reprogramming also remains a dominant feature of immune evasion. BrM secretes signals that redirect monocyte-derived macrophages and microglia toward immunosuppressive phenotypes, which show patterns of gene expression dominated by type 1 interferon (IFN) activity and checkpoint signaling (9). This means that they are wired into a more immunosuppressive state that promotes T cell dysfunction, rather than anti-tumor defense. These signatures were more enriched in BrM-derived microglia compared to glioma-derived microglia (9). This difference underscores how metastases actively reshape the CNS immune landscape, allowing malignancies to persist and proliferate. In conjunction, the BrM microenvironment also contains lots of macrophages and exhausted CD8**^+^** T cells, with context-dependent mechanistic contributions from B cells, plasma cells, and Tregs, which all help the tumor evade immune attack (9).

**Figure 2:**
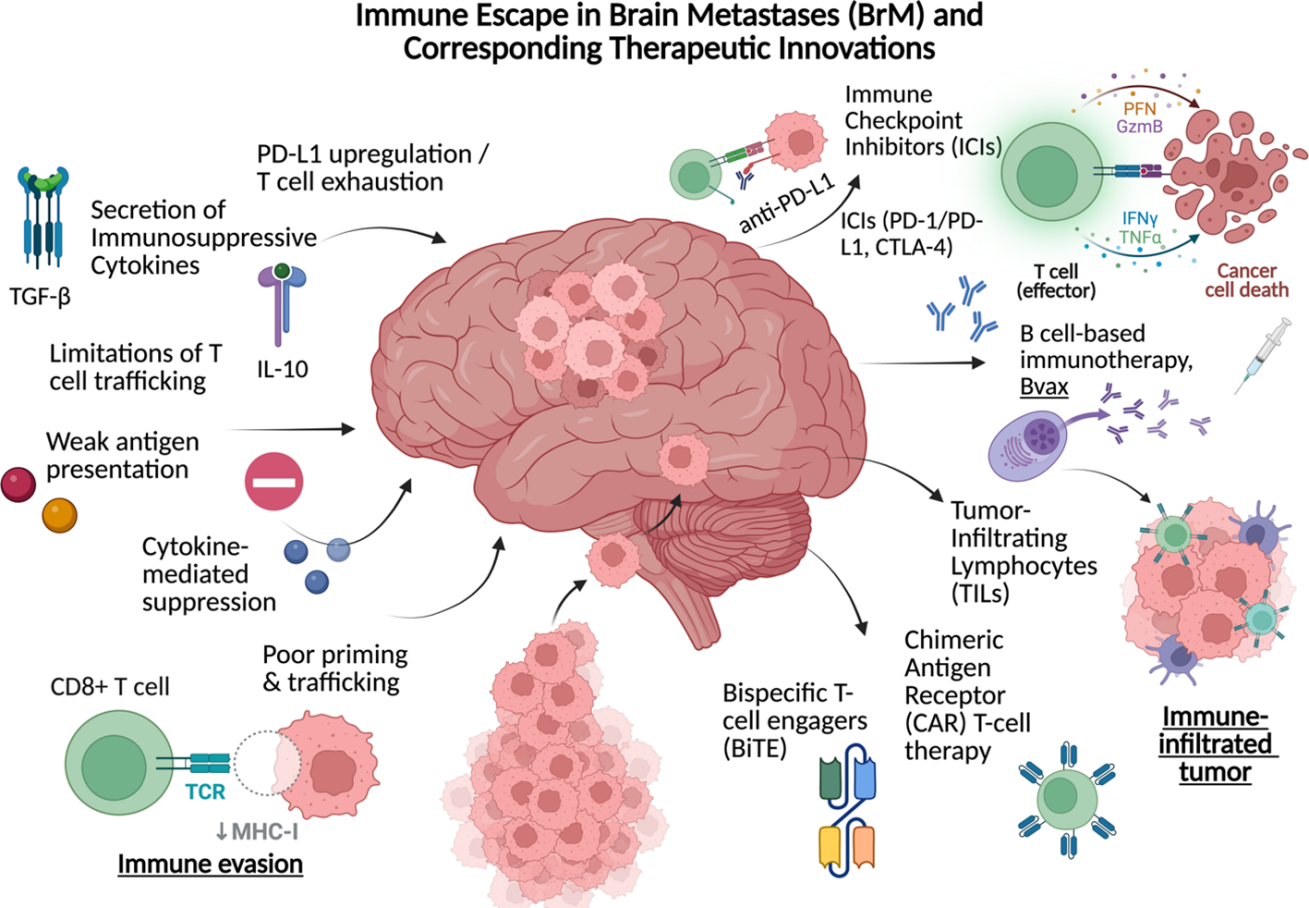
Detailed immune evasion mechanisms that allow metastatic development partnered with corresponding novel therapeutic strategies and developments.

Collectively, the immune escape mechanisms including checkpoint upregulation, cytokine cross-talk, and myeloid remodeling produce an immune-excluded tumor milieu. These mechanisms provide important insights into the logic of modern therapy approaches. For instance, checkpoint blockade seeks to reverse T cell dysfunction, adoptive T cell (ACT) and CAR T- cell therapy, a subset of ACT, aim to bypass trafficking and exhaustion barriers (101). In addition, B cell-based immunotherapies attempt to restore antigen presentation and antibody-driven immunity. So, the features that permit the BrM persistence have ultimately become the therapeutic targets shaping current CNS-directed immuno-oncology (**Figure 2**).

## 5 Recent immunotherapeutic approaches

As there are limited treatment options available for patients with BrM, a critical question is whether a better understanding of how primary and metastatic malignancies develop and interact within the brain TME can reveal novel therapeutic targets (69). A few immunotherapeutic strategies have emerged with recent studies, including ICIs, TIL therapies, bispecific T-cell engagers (BiTEs), CAR T-cell therapies, and vaccine-based approaches. Increasing preclinical and clinical evidence supports the use of these therapies in the BrM context.

### 5.1 Efficacy of immune checkpoint inhibitors

The discovery of immune checkpoint pathways has marked a shift in the cancer immunotherapy realm. Currently, the U.S. FDA has approved three major classes of ICIs including programmed cell death protein 1 (PD-1) inhibitors (Nivolumab, Pembrolizumab, Cemiplimab), programmed death-ligand 1 (PD-L1) inhibitors, and the cytotoxic T-lymphocyte-associated protein 4 (CTLA-4) inhibitor (Ipilimumab) (102). These mechanisms dampen T cell activation, and the inhibitory receptors enable tumors to evade immune surveillance; so, blocking them restores and enhances anti-cancer immune (102). In the preclinical setting, previous studies utilized the CMT167 and LLC murine models of lung adenocarcinoma with high brain tropism, demonstrating that anti-PD-1/PD-L1 therapies could present a possible avenue for treatment of (103, 104). This anti-PD-1/PD-L1 therapy effectively reduced growth of primary orthotopic tumors, with up to 95% inhibition of CMT167 orthotopic tumor growth. There was also 30% inhibition of CMT167 subcutaneous tumors, and 35% inhibition of LLC orthotopic lung (103). With that being said, the brain tropic tendencies of cell lines could be harnessed to evaluate the efficacy in the BrM. In a tangential study, combination where ICIs, like anti-PD-1/PD-L1, are utilized in conjunction with an intratumoral injection of immune-activating agent (such as oncolytic viruses, and agonists), yielded synergistic anti-tumor effects, most likely caused by increased T cell infiltration and reduced (104). This was an attempt to convert “cold” tumors into “hot” immunologically responsive tumors. Mechanistically, these therapies re-energized exhausted immune cells within the brain TME, helping to facilitate cytotoxic (104, 105).

ICIs have also revolutionized treatment in the clinical setting. This is particularly seen in NSCLC patients afflicted with general metastases, which could show promise for BrM patients. For instance, the KEYNOTE-189 phase 3 trial probed the use of pembrolizumab, a PD-1 inhibitor, in combination with a platinum-based chemotherapy as a first- line treatment (106). There were improvements in overall survival (OS) and progression-free survival (PFS) over chemotherapy alone. CNS response rates were approximately 29.7% in PD-L1-positive patients. Many patients were able to avoid radiation treatment to the brain, and greater than 33% of the patients survived at least two years after the start of the clinical trial (106, 107). Additionally, the CheckMate 227 trial evaluated nivolumab (anti-PD-1) in combination with ipilimumab (anti-CTLA-4) in advanced NSCLC, including those with baseline treated brain metastases (108, 109). At a minimum follow-up of 5 years, BrM patients who received the combination ICI therapy (chemotherapy + ICI) had a median overall survival of 17.4 months compared with 13.7 months for solely chemotherapy (hazard ratio = 0.63), and demonstrated higher 5-year systemic (12% vs. 0%) and intracranial progression-free survival rates (16% vs 6%), which indicates improved long-term systemic and intracranial disease control (109).

### 5.2 Tumor-infiltrating lymphocyte therapy

The basis of TIL therapy involves isolating T cells from patient tumors, expanding them ex vivo, and reinfusing them to target malignancies. Its efficacy was first demonstrated in foundational melanoma trials, contributing to the development of adoptive cell therapy for solid tumors. Approval of lifileucel (Amtagvi) in February 2024 marked the first FDA-approved tumor-derived T-cell therapy for advanced melanoma, paving the way for its evaluation in preliminary contexts like BrM (110, 111). In the pivotal clinical study, phase 2 of C-144–01 trial published in 2022, lifileucel achieved systemic disease control, with a 31.4% objective response rate (ORR), including eight complete and 40 partial responses, with a median response duration exceeding three years; approximately one-third of responders remained progression-free at five years (112, 113). Although hematologic toxicity was experienced, responses to lifileucel were durable in a heavily pretreated population, where 81.7% of the trial population were pretreated with anti-PD-1 and anti-CTLA-4 agents (110).

Early-phase trials suggest that TIL therapy may also have activity in patients with CNS involvement. In the adoptive TIL therapy trial NCT02360579, lifileucel produced encouraging intracranial responses, some BrM cases, with minimal toxicity in metastatic melanoma (114). In addition, NCT03083873 was a phase 2 C-145–03 trial that evaluated autologous TIL therapy (LN-145), specifically in patients with recurrent or metastatic head and neck squamous cell carcinoma (HNSCC) (115, 116). Patients had surgical tumor resection to generate LN-145, followed by non-myeloablative lymphodepletion (NMA-LD) and IL-2 administration (115). Among the 52 patients treated, the ORR was 11%, the disease control rate was 76%, median OS was 9.5 months, and some responses, regarding systemic disease control, persisted beyond 23 months despite the heavily pretreated cohort (115). Collectively, these findings indicate that TIL therapy can harness durable antitumor activity systemically, and may retain functions within the CNS, supporting a preliminary relevance for BrM, although further intracranial studies remain limited compared to those of extracranial disease trials.

### 5.3 Emerging immunotherapies

#### 5.3.1 Bispecific T-cell engagers

In an attempt to bypass the limitations of endogenous T cell priming and infiltration or typical antigen presentation, bispecific T-cell engagers (BiTEs) link cytotoxic T cells to TAAs by engaging CD3 and a tumor antigen (117, 118). This mechanism is particularly intriguing in the BrM realm where ICI efficacy is restricted due to local immunosuppression and the limitation of T cell infiltration.

A promising target for BiTEs is delta-like canonical Notch ligand 3 (DLL3), a protein aberrantly expressed in varying tumors, especially in small-cell lung cancer (SCLC) (119), a disease with high BrM incidence. The DLL3-directed BiTE tarlatamab (AMG 757) was shown to harness T-cell activation and cytotoxicity and has demonstrated antitumor activity in early clinical trials, including patients with stable BrM (120–122).

As seen in the phase 1 AMBER trial (NCT03319940), tarlatamab produced an ORR of 23.4% with the median duration of response being 12.3 months, PFS 3.7 months, and median OS 13.2 months in relapsed or refractory SCLC, regarding systemic disease control (121). The results of this study suggested that DLL3 had potential to be a viable clinical target, despite manageable toxicity noted. Furthermore, the phase 2 DeLLphi-301 (NCT05060016) shows preliminary promise in tackling the presence of BrM in small-cell lung cancer (SCLC). With approximately 40-70% of patients afflicted with SCLC developing brain metastases, this trial assesses tarlatamab effectiveness to mitigate or promote regression of patients with stable, asymptomatic BrM (122, 123). 29% of the trial cohort (54/186) had treated and stable BrM, and the majority of patients had received previous local radiotherapy. Overall, the systemic ORR was 45.3% in patients with BrM and 32.6% in patients without BrM (122). From this, tarlatamab revealed preliminarily promising efficacy along with a positive benefit-risk profile in patients with pre-treated SCLC, including patients with stable BrM. The findings can suggest that BiTEs, specifically the DLL3-targeted constructs, could be a convincing alternative to other therapies like TIL or ICI. However, challenges still remain for utilizing in BrM such as antigen heterogeneity, toxicity, and uncertainty regarding CNS responses, especially with a pool of treatment-naïve BrM patients.

#### 5.3.2 Harnessing chimeric antigen receptor T-cell therapy in BrM

CAR T-cell therapy redirects T cells to recognize and eliminate malignant cells through an engineered antigen-binding domain, fused to intracellular costimulatory and signaling motifs. The recognition domain allows for the T cell to identify and act upon cancer cells. While CD19-directed CAR T-cell therapies revolutionized treatment of hematological malignancies (123), translating this success to solid tumors, and especially into the CNS, has posed more of a challenge due to antigen heterogeneity, the immunosuppressive TME, and the restrictive BBB.

In an orthotopic murine BrM model with EpCAM-expressing lung carcinoma, Xu et al. demonstrated that CAR T-cell therapy efficacy was highly dependent on delivery route (124). Local administration enabled tumor infiltration, IFN-γ production, tumor cell lysis, and tumor regression, in addition to prolonged survival, whereas intravenous delivery resulted in minimal intratumoral trafficking and limited therapeutic benefit (124). This suggests the delivery route as a key determinant of CAR T-cell therapy efficacy in BrM. Intratumoral persistence of CAR T-cell therapy following one dose remained limited, which signaled durability as a main concern (124). Other preclinical studies showed epidermal growth factor receptor (EGFR), a commonly altered oncogene in GBM and also expressed in subsets of NSCLC and breast cancers giving rise to BrM, represents a potential CAR T-cell therapy target, in addition to EpCAM, which has been implicated within the metastatic cascade and in maintaining tumor stemness or orchestrating self-renewal (125). EGFRvIII could act as an even more attractive CAR T-cell target (126), and both EpCAM and EGFRvIII may be relevant in the BrM context due to potential to cross the BBB, as suggested in xenograft models (127), as these may be upregulated and maintained during metastatic progression (128, 129). The use of this EGFRvIII-targeted CAR T-cell therapy, with prior resection or radiotherapy, showed substantially increased survival and decreased lung tumor burden in preclinical murine models (130), in addition to metastasis of EGFRvIII-positive lung cancer cells in mice being significantly inhibited and, regarding systemic disease control, OS was increased, while avoiding toxicity or adverse effects (130, 131).

Further *in vivo* success has driven early clinical translation of engineered CAR T-cell therapies. In a recruiting phase 1 study (NCT0506796), with intended advanced-stage NSCLC patients, CXCR5-modified CAR-T cells will be tested against EGFR (132). These CAR T-cells are engineered to co-express the chemokine receptor CXCR5, which facilitates their migration toward CXCL13-rich tumor sites and enhances intratumoral trafficking and local cytotoxicity (133), overcoming limited T cell infiltration across the BBB. A tangential study (NCT04153799) has similar targets and is optimizing the doses of EGFR-targeting CAR T-cells in conjunction with assessing potential adverse events, with reported grade 1–2 toxicities (131, 134).

In addition to the EGFR-targeted avenue, CD133-directed CAR T therapy has recently presented as a potential BrM- relevant approach. CD133, expressed in many solid tumors, is a glycoprotein that marks tumor-initiating cell populations associated with resistance and metastasis. In lung and colon cancer-derived BrM patient xenograft models, a single intratumoral dose of CD133-CAR T cells induced strong tumor regression, prolonged survival, and complete remission was noted in patients afflicted with colon cancer BrM (135, 136). Altogether, the aforementioned preclinical and early clinical studies demonstrate the feasibility of targeting EGFR/EGFRvIII, EpCAM, and CD133 to improve CNS penetration, address tumor-initiating cell subsets, and potentially enhance the durability of CAR T-mediated control of BrM. While encouraging, these studies remain preliminary and largely hypothesis-generating, underscoring the need for further clinical evaluation and exploration to define the true potential of such therapies in BrM. The limitations of these approaches suggest an opportunity to explore alternative therapies which may offer complementary advantages, such as B cell-based immunotherapies, which could enhance trafficking, sustain antibody production, and immunological memory within the CNS.

#### 5.3.3 Vaccine-based approaches

While CAR T-cell therapies focus predominantly on the transfer of engineered lymphocytes, cancer-based vaccines work to educate the patient’s immune system to distinguish and attack malignant cells. For BrM in particular, vaccine approaches are compelling as they could provide durable immune surveillance against varying and disseminating tumor antigens that drive recurrence. Preclinical and clinical findings have suggested that vaccines are able to produce effective anti-tumor immunity even amid the immunosuppressive CNS microenvironment. Mechanistically, these approaches encompass peptide-based vaccines targeting TAAs, dendritic cell (DC)-based vaccines loaded with patient-derived tumor lysates, and neoantigen-based approaches designed from varying individualized mutational patient profiles (137).

Notable dendritic cell-based vaccine strategies that have compelling findings include the DCVax-L platform (NCT00045968) and the PerCellVax3 trial (NCT02808416) (138, 139). The DCVax-L platform (Northwest Biotherapeutics) is an autologous dendritic cell vaccine that is loaded with tumor lysate, extensively tested in GBM patients. Despite it being less BrM-focused, this still provides details regarding the intracranial response and potential efficacy in other BrM malignancies originating from extracranial tumors. The prognosis for GBM patients remains poor, as recurrence is a common tendency after surgery and radiation therapy, while median survival remains dismal. Phase I and II clinical trials were conducted at UCLA with DCVax-L for combatting the immunologically cold GBM. The trials consisted of 39 patients, 20 with newly diagnosed GBM while 19 had recurrent GBM. Newly diagnosed patients receiving DCVax, with standard of care treatment, did not experience tumor recurrence for approximately 2 years compared to typical recurrence tendency of 7 months, and had a median survival of 3 years (138, 140, 141). A significant number of patients within these early-phase trials actually persisted in a “long tail” survival, even extending beyond the 3-year median survival. 33% were noted to exceed 4 years, and 27% had reached 6 years’ survival, showing the promise of this DC-based approach and administration to patients.

In parallel, the PerCellVax3 trial tested the efficacy of a personalized cellular vaccine for patients afflicted with brain metastases. These vaccines consisted of dendritic cells that were pulsed with mRNAs encoding tumor antigens, rather than with bulk tumor lysate. As established in the Jinan University Affiliated Guangdong 999 Brain Hospital, the personalized TAA-based DC vaccines were tested in trials of some patients with GBM (NCT02709616), recurrent GBM (NCT02808364), and lung cancer with brain metastasis patients (NCT02808416) (142–145). The median survival was 17 months for the lung cancer patients with BrM and 19 months for GBM patients, compared to median OS of approximately 6–10 months for patients receiving only standard-of-care treatment (such as chemotherapy or radiotherapy) (142). All five NSCLC patients demonstrated partial response (PR) when evaluated 3 months after the start of immunotherapy. Throughout the trial, it was noted that 13 NSCLC with BrM patients and 28 GBM patients were administered standard treatment (RT/chemo/surgery) in combination with the DC vaccine. Those that received immunotherapy had a more promising outcome compared to patients solely receiving standard-of-care therapy (142). Overall, there was strong induction of antigen-specific CD4**^+^** and CD8**^+^** T cell responses to most TAAs within the vaccine panels. Within the BrM samples, several TAAs such as MUC1, IGF2BP3, and STAT3 were intensely upregulated. In particular, IDO1 was the dominant, overexpressed, immunosuppressive factor in the lung cancer brain metastatic samples. Although TAA expression was heterogeneous across tumors, the recurrent involvement of STAT3- and IDO1-driven pathways underscores their roles in tumor proliferation, immune evasion, and metastatic progression (142, 146, 147). These findings demonstrate that the personalized, multi-TAA DC-based vaccines are feasible in BrM patients, with intracranial and systemic responses noted, especially in combination with other standard treatments or checkpoint blockade. In all, the researchers emphasized the small trial cohort of this study, and the need for larger, randomized studies in efforts to attain confirmation of efficacy and explore potential nuances.

One promising avenue is B cell-based immunotherapy (B_Vax_), which has been curated to selectively amplify pro-inflammatory, antigen -presenting B cell s. Ex vivo stimulation with CD40 agonism and cytokines, followed by loading tumor antigen, propels B cells toward an immunostimulatory phenotype. This can be characterized by enhanced antigen presentation, costimulatory ligand expression, and cytokine secretion (148, 149). These engineered B cells function as alternative professional antigen presenting cells (APCs), which directly prime CD4**^+^** and CD8**^+^** T cells. Evaluated in murine glioma models using GL261-OVA and CT2A cell lines, BVax significantly enhanced tumor-infiltrating CD8**^+^** T cells (>2-fold increase compared to naïve B cells) and induced elevated expression of GranzymeB and IFN-γ within intracranial tumors (148, 149). In CT2A-bearing mice, BVax combined with standard-of-care treatment, such as radiotherapy, and PD-L1 blockade conferred tumor eradication in 80% of the animals, while also promoting immunological memory in order to prevent tumor recurrence (148). In GBM patient-derived BVax, *in vitro* experiments showed success in activating autologous CD8**^+^** T cells, which were able to eradicate glioma cells, and emphasized the translational potential of this approach (148). The parallels of gliomas or primary tumors with BrM, like barrier disruption, myeloid dominance, and immune exclusion, present a strong rationale for translation to treatment of brain metastases. BrM tend to retain antigenic heterogeneity from extracranial primary malignancies (78), which provides further opportunities for BVax to broaden T cell recognition (**Table 1**).

**Table 1:** Summary of preclinical and clinical trials across different immunotherapy modalities, relevant to BrM.

Trial/Study	Therapeutic agent	Phase	Indication	Outcome summary	Reference
Preclinical, CMT167, LLC models	Anti-PD-1/Anti-PD-L1	Preclinical	Lung adenocarcinoma (brain-tropic models)	95% inhibition of CMT167 orthotopic growth; 30-35% inhibition in s.c. or LLC models	(103, 104)
KEYNOTE-189	Pembrolizumab + platinum chemo	Phase 3 clinical trial	Metastatic NSCLC	Improved OS and PFS vs chemo; >33% survival at ≥2 years; many avoided brain RT; CNS response ~29.7% in PD-L1+	(106)
CheckMate 227	Nivolumab + Ipilimumab	Phase 3 clinical trial	Advanced NSCLC with baseline treated BrM	Median OS 17.4 vs 13.7 months; improved long-term systemic control & Intracranial; 5-yr intracranial PFS: 16% vs 6%	(108, 109)
C-144-01 (NCT02360579)	Lifileucel	Phase 2 clinical trial	Metastatic melanoma	ORR 31.4%; median response >3 yrs; ~1/2 progression-free at 5 yrs; systemic responses durable	(112, 113)
C-145-03 (NCT03083873)	LN-145 TIL therapy	Phase 2 clinical trial	Recurrent/metastatic HNSCC	ORR 11%; disease control 76%; median OS 9.5 mo; durable response in pretreated patients	(115, 116)
AMBER (NCT03319940)	Tarlatamab (DLL3 BiTE)	Phase 1 clinical trial	Relapsed/refractory SCLC	ORR 23.4%; DOR 12.3 mo; PFS 3.7 mo; OS 13.2 mo	(121)
DeLLphi-301 (NCT05060016)	Tarlatamab	Phase 2 clinical trial	SCLC, including stable BrM	ORR 45.3% (BrM+) vs 32.6% (BrM-)	(122)
EpCAM-CAR-T	EpCAM	Preclinical	Lung cancer, including patients with BrM	Local delivery prolonged survival; IV delivery not as effective; BBB penetration in xenografts	(124)
EGFR/EGFRvIII CAR-T	EGFR/EGFRvIII	Preclinical	NSCLC, breast cancer, GBM	Tumor regression; improved survival with low toxicity	(128, 132. 154)
NCT0506796	CXCR5-modified EGFR CAR-T	Phase 1 clinical trial	Advanced NSCLC	CXCR5 enhances migration to CXCL13-rich brain TME	(132–134)
NCT04153799	EGFR-CAR-T	Phase 1 clinical trial	Solid tumors	Grade 1–2 toxicities; Intracranial tumor regressions	(130, 131)
CD133-CAR-T	CD133	Preclinical	Metastatic lung and colon cancer, including BrM	Single intratumoral dose induced strong regression; complete remissions in colon BrM patients	(135, 136)
DCVax-L	Autologous DC vaccine with tumor lysate	Phase 1–2 clinical trial	Diagnosed and recurrent GBM	Median survival ~3yrs; many patients lived ≥4–6 yrs; delayed recurrence	(138, 141)
PerCellVax3 (NCT02808416)	Personalized DC vaccine with mRNA-encoded TAAs	Early Phase clinical trial	Lung cancer with BrM; GBM	NSCLC BrM median OS 17 mo; GBM 19 mo; NSCLC BrM pts PR at 3 mo; strong CD4/CD8 induction	(142–145)
BVax (GL261-OVA, CT2A models)	CD40-activated, cytokine-stimulated, antigen-loaded B cells	Preclinical	GBM (BrM-relevant mechanisms)	>2 fold CD8**^+^** infiltration in CNS; 80% tumor eradication with RT + PD-L1 blockade; memory induction; potent APC activity	(148, 149, 172)

## 6 Limitations of current immunotherapies

Despite the encouraging preclinical and clinical evidence of the efficacy of ICIs, adoptive cell therapies, bispecific constructs, CAR T, and vaccine-based approaches, there are still challenges in navigating the tumor microenvironment and CNS vasculature. Limitations remain that restrict the widespread adoption of these strategies, especially for targeting BrM.

### 6.1 Heterogeneity of antigen expression

A recurring obstacle entails that of heterogeneity, inter- and intralesional, across BrM development. While the aforementioned targets, including PD-L1, EGFR/EGFRvIII, EpCAM, DLL3, and CD133, have shown relevance and promise in preclinical or clinical studies, the expression of these targets is often unfortunately transient. Tumors often “outsmart” the targeted immunotherapy. The antigen variability could be responsible for the development of adaptive resistance, essentially the tumor’s ability to adapt dynamically to the immune attack. Surviving tumor subclones are those that do not express the antigen, lack the therapeutic target, and expand or repopulate under immune pressure. For instance, in the case of EGFRvIII-directed CAR-T therapy, the loss of antigen expression was a prominent mechanism of recurrence in GBM, which raised similar concerns for the BrM context (126, 150, 151).

### 6.2 TME-mediated immunosuppression

As the CNS TME is characterized as immunosuppressive, it is shaped by the interplay of blood-brain-barrier (BBB) restrictions, reduced T cell infiltration, and increased expression of suppressive inhibitory molecules, like IDO1, TGF-β, and STAT3 (142, 146, 147). This landscape continues to pose a challenge for therapeutic efficacy. Previously, the CNS was seen as “immune-privileged” because of the restrictive blood-brain and blood-CSF barriers, which presents a wall to entry of peripheral immune cells. However, recent discoveries of CNS lymphatic pathways and border-associated immune niches, like meninges and choroid plexus, have challenged this ideology, highlighting that brain malignancies aren’t entirely sequestered from systemic immunity (152).

Within BrM, tumor infiltration bypasses and disrupts the BBB, allowing the recruitment of peripheral immune cells, but also signaling chronic inflammation. The resident CNS cells, like astrocytes, microglia, and neurons, interact dynamically with infiltrating myeloid or lymphoid cells in efforts to curate an immunosuppressive niche. For example, the reactive astrocytes and microglia tend to release immunomodulatory factors, including TGF-β, IL-10 or VEGF, that weaken cytotoxic T cell activity while also promoting angiogenesis and tumor growth. Tumor-related macrophages and recruited myeloid-derived suppressor cells essentially foster this inhibitory environment, essentially pushing towards T cell exhaustion and diminished persistence (76, 152, 153).

This immunosuppressive crosstalk not only protects the metastatic cells from immune-mediated destruction, but also fosters metabolic stress, reduces antigen presentation, and accelerates therapeutic resistance. Notably, while TILs are often correlated with a more optimistic prognosis and immunotherapy response, in brain metastases, myeloid-lineage immune cells are found more predominantly compared to tumor-filtrating cytotoxic T cells. Rather attacking the tumor, the myeloid cells tend to adopt a more pro-tumor and immunosuppressive function, which fosters metastatic progression and therapeutic resistance (152). Therefore, the brain metastatic niche isn’t simply a passive sanctuary, rather serving as an active contributor in suppressing effective anti-tumor immunity. This poses a central barrier to harnessing a durable response across CAR-T therapy, ICI, and vaccine-based approaches.

### 6.3 CNS barriers and delivery dependence

The sequestered nature of the CNS vasculature, particularly the BBB and BTB, remain significant hurdles for adequate systemic therapy delivery. While locoregional strategies, like intraventricular and intratumoral delivery, help to improve trafficking, these approaches can be rather invasive and pose a concern for feasibility if patients have inaccessible or multifocal metastases. For instance, this delivery dependence was observed with the intravenous infusion of EpCAM or EGFR-directed CAR-T cells which yielded negligible intratumoral infiltration in preclinical murine BrM models, while local delivery was effective but lacked durability with a single dose (79).

### 6.4 Durability and response persistence

Immunotherapy responses in BrM tend to be transient, with tendencies of relapse because of immune evasion or inadequate immunological memory formation. Vaccine-based platforms like DCVax-L or PerCellVac3 induce robust CD4**^+^** and CD8**^+^** T cell responses, as detailed previously, although their durability in the CNS remains unclear, especially given the immunological “coldness” of the brain and associated malignancies. Similarly, BiTEs like DLL3-targeted tarlatamab display optimistic efficacy in SCLC with BrM, but responses are often short-lived without continued infusion. In the tarlatamab trial, the majority of the patient cohort was also heavily pre-treated, which could have played into the effects and nuances of the immunotherapy application (121).

### 6.5 Toxicity implications and limited BrM representation

Although mostly tolerable, immunotherapies can induce side effects and potential severe neurological toxicities within the BrM context. With the application of ICIs, there have been instances of exacerbated cerebral edema and increased necrosis risk when in combination with stereotactic radiosurgery (SRS). Seizures were an initial symptom in a striking 40% of melanoma brain metastatic (MBM) patients (154), which may also be aggravated by ICIs, resulting in the need for antiepileptic drug usage during some trials (155, 156). Symptomatic edema was also reported in the CheckMate-204 trial in particular, with incidence ranging up to 36% with combined ipilimumab and SRS in melanoma-derived brain metastases (157, 158). Baseline edema often does not impact anti-PD-1 response in melanoma or NSCLC patients (159), although symptomatic edema often requires ICI interruption, corticosteroids, and supplementary local therapy including surgery or radiation treatment (156). Corticosteroids are known to be widely used in BrM, and patients with primary brain tumors, for symptomatic relief of intracranial pressure or edema, with recommended doses starting at 4–8 mg/day of dexamethasone and potential escalation to ≥16 mg/day for optimal mass effect (160, 161). Baseline or high-dose systemic steroid use has been associated with unfortunately reduced ICI efficacy and lower PFS/OS in metastatic NSCLC and melanoma, as suggested by a meta-analysis in BrM patients with reported shorter systemic PFS (Hazard Ratio ~2.0) and worse OS (Hazard Ratio ~1.84) with steroid use (160, 161). Corticosteroids are also utilized in patients with GBM for edema control, and their effects are correlated to reducing vasogenic edema rather than exerting antitumor activity (162). Additionally, CAR-T and BiTE cell therapies can trigger and induce cytokine release syndrome (CRS). CRS is a cascade of toxic adverse events seen during infection or after antibody administration for therapeutic testing. It is a response associated with the high circulating concentrations of different pro-inflammatory cytokines, like stimulating factors, necrosis factors, and more (163, 164). There have been recent efforts to prevent and control CRS, although it is still an unmet clinical need.

With the plethora of studies analyzed above, BrM patients remain still underrepresented in clinical trials. Many pivotal studies exclude those with symptomatic and untreated CNS metastases, which poses a concern for widespread efficacy. Current evidence originates from trials with smaller cohorts, fewer randomized trials, *post-hoc* analyses, or from trials that are in the exploratory stage. This poses a major concern in generalizing findings for treatments tailored to patients afflicted with brain metastases.

## 7 Perspective: role of B cells in cancer immunity and B cells in BrM context

B cells, long considered as antibody-producing effectors of humoral immunity, have more recently been acknowledged as multifaceted and context-dependent regulators of anti-tumor immunity (165–167). Beyond the generation of immunoglobulins, B cells serve several vital functions such as antigen presentation, cytokine secretion, facilitation of the adaptive immune responses, and maintenance of TLS, which are associated with enhanced anti-tumor responses and improved outcomes with ICIs in varying solid tumors (11). However, in the realm of cancer, it is important to acknowledge that contemporary mechanistic and clinical studies underscore that B cell activity is not entirely pro-response, as it is profoundly context-dependent and varies depending on differentiation, isotype class switching, cytokine milieu, and TLS maturation or composition (165). B cell subsets such as Bregs, IL-10 producing phenotypes, and immature TLS composition can exert a more immunosuppressive (pro-tumor) effect, whereas class-switched memory B cells, plasma cells, and germinal-center-like programs tend to link to anti-tumor responses (168). Delving into this duality is vital in the context of BrM, where the immune landscape is already influenced by the niche constraints of the CNS. Thus, with an accumulation of immunotherapeutic response evidence suggesting the anti-tumor B cell tendencies, it is important to acknowledge this heterogeneity in addition to the immunological pressures intracranially.

One of the most renowned contributions of B cells to tumor immunity is the production of tumor-specific antibodies. Antibodies can bind tumor antigens and mediate effector functions through antibody-dependent cellular cytotoxicity or complement -dependent cytotoxicity, thereby facilitating the recruitment of NK cells, macrophages, and other such effector populations to promote tumor cell destruction (169, 170). Antibody opsonization, or the flagging of tumor cells, also enhances the engulfing of tumor material by APCs. This allows for the harnessing of cross-presentation of tumor antigens and creating durable adaptive responses with priming of T cell responses.

Equally important is the function of activated B cells as professional APCs. B cells can ultimately prime both CD4**^+^** and CD8**^+^** T cells with the upregulation of MHC class I and II molecules in addition to costimulatory ligands, like CD80 and CD86 (171). This unique dual role positions B cells as key mediators in bridging innate and adaptive immune compartments, which have been therapeutically harnessed like in B cell-based immunotherapy, such as BVax (172). This approach harnesses ex vivo-activated B cells activated with tumor antigens in efforts to enhance CD8**^+^** T cell priming, which is one of the many functions of BVax.

Beyond the role of antigen presentation, B cells have an influential effect through their cytokine repertoire/diverse collection. Some activated effector B cells can ultimately produce and secrete pro-inflammatory mediators like IFN-γ, IL-12, and TNF-α, which enhance cytotoxic T cell and NK cell activity (173). Adjacently, subsets of Bregs secrete immunosuppressive cytokines like IL-10 and TGF-β, which tend to attenuate effector immunity while promoting regulatory T cell expansion and facilitating tumor immune invasion (174). Recent mechanistic analyses further emphasize the therapeutic plasticity of B cells. The activation of 4-1BBL^+^ B cells with CD40 agonism and IFN-γ stimulation has been correlated to amplification of the antigen-presenting capacity, therefore skewing cytokine production towards a more pro-inflammatory phenotype, and ultimately supporting robust CD8**^+^** T cell responses (148). This suggests that the balance between effector and regulatory B cell subsets, including the cytokine outputs, critically influences the route of anti-tumor immunity, presenting nuanced challenges and opportunities for the utilization of therapeutic intervention in targeting brain metastases.

A striking feature of B cells in the cancer context is their localization within TLS. B cells facilitate and coordinate with T cells, dendritic cells, and follicular helper T (Tfh) cells in order to sustain antigen presentation and promote affinity maturation as well as durable memory. Across solid tumor malignancies, TLS are deeply associated with improved prognosis and enhanced responsiveness to immune checkpoint blockade treatments. TLS presence in BrM underscores their role as concentrated centers of anti-tumor immunity. However, the activity is context dependent. BrM are influenced by immunosuppressive myeloid cells and reactive astrocytic interactions, which push infiltrating B cells toward regulatory phenotypes. The Bregs suppress CD8**^+^** T cells, expand Tregs, and further promote M2 macrophage differentiation, collectively reinforcing tumor progression (175, 176). Thus, this duality of B cell contributions presents both an opportunity and challenge for therapeutic intervention.

Through integrating checkpoint blockade with innovative treatment delivery approaches, B cell-based immunotherapy approaches, such as BVax, may form a promising axis of immune activation, used synergistically with other approaches, in order to target brain metastases. This would broaden the current therapeutic landscape for BrM beyond T cell-centric strategies and harness the emerging potential of B cells in immunotherapy.

### 7.1 Concluding remarks

B cell-based immunotherapies have the possibility to overcome several limitations of T cell-focused approaches for treating BrM. T cells in the CNS often exhibit notable functional exhaustion, limited antigen access, and hindered recruitment because of the BBB and immunosuppressive astrocyte-myeloid networks (177). However, B cells offer complementary functions such as local antigen presentation, intratumoral antibody production, cytokine polarization, and facilitation of TLS formation which could circumvent some of these recognized barriers and present therapeutic opportunity. Preclinical work with BVax and 4-1BBL+/CD40-activated B cells, as discussed previously, shows that ex vivo-B cells can drastically enhance CD8**^+^** T cell priming, encourage cytotoxic responses, and shape suppressive niches (148, 149). These insights show promise for B cells as a possible synergistic partner capable of improving checkpoint efficacy or restoring activity in immune-restricted BrM. It is important to acknowledge that translational challenges remain, especially due to BrM being often in accessible, unresected, or biopsy-limited, which restricts high-resolution profiling of spatially organized B cell states, TLS formation, and Breg-influenced niches (178). Circulating B cell markers provide promise, but not the whole picture, of understanding the role of B cells within BrM and the CNS immune environment. Within BrM, infiltrating B cells, such as naïve, plasma, and memory B cells, in tumors are positively correlated with patient prognosis, and the overarching heterogeneity of subpopulations provides insight into the degree of anti-tumor response (42). The presence, density, and maturation of TLSs have also been demonstrated to be notable prognostic markers (42). Overall, this review denotes a vital therapeutic gap in BrM management and proposes leveraging B cells for treatment as a promising avenue to improve the prognosis of BrM-afflicted patients.

## 8 Methods

A literature search was conducted using PubMed to source relevant studies on the current immunotherapies for BrM and leveraging B cell functions for treatment of BrM arising from primary malignancies. Search terms included keywords and MeSH terms such as “B cells,” “immunotherapy,” “dual role of B cells,” “tertiary lymphoid structures,” “brain metastases,” “brain tumor microenvironment,” and “brain metastases treatments.”

The search was mostly limited to articles between 2010-2025. Original research articles and relevant reviews were included to ensure comprehensive coverage of topics.
